# A Simple Procedure for Creating Scalable Phenotypic Screening Assays in Human Neurons

**DOI:** 10.1038/s41598-019-45265-1

**Published:** 2019-06-21

**Authors:** BanuPriya Sridharan, Christopher Hubbs, Nerea Llamosas, Murat Kilinc, Fakhar U. Singhera, Erik Willems, David R. Piper, Louis Scampavia, Gavin Rumbaugh, Timothy P. Spicer

**Affiliations:** 1 The Scripps Research Molecular Screening Center, Department of Molecular Medicine, Scripps Research, Jupiter, Florida 33458 USA; 2 Department of Neuroscience, Scripps Research, Jupiter, Florida 33458 USA; 3 Graduate School of Chemical and Biological Sciences, Scripps Research, Jupiter, Florida 33458 USA; 4 Cell Biology, Thermo Fisher Scientific, Carlsbad, California 92008 USA

**Keywords:** Phenotypic screening, Stem cells in the nervous system

## Abstract

Neurons created from human induced pluripotent stem cells (hiPSCs) provide the capability of identifying biological mechanisms that underlie brain disorders. IPSC-derived human neurons, or iNs, hold promise for advancing precision medicine through drug screening, though it remains unclear to what extent iNs can support early-stage drug discovery efforts in industrial-scale screening centers. Despite several reported approaches to generate iNs from iPSCs, each suffer from technological limitations that challenge their scalability and reproducibility, both requirements for successful screening assays. We addressed these challenges by initially removing the roadblocks related to scaling of iNs for high throughput screening (HTS)-ready assays. We accomplished this by simplifying the production and plating of iNs and adapting them to a freezer-ready format. We then tested the performance of freezer-ready iNs in an HTS-amenable phenotypic assay that measured neurite outgrowth. This assay successfully identified small molecule inhibitors of neurite outgrowth. Importantly, we provide evidence that this scalable iN-based assay was both robust and highly reproducible across different laboratories. These streamlined approaches are compatible with any iPSC line that can produce iNs. Thus, our findings indicate that current methods for producing iPSCs are appropriate for large-scale drug-discovery campaigns (i.e. >10e^5^ compounds) that read out simple neuronal phenotypes. However, due to the inherent limitations of currently available iN differentiation protocols, technological advances are required to achieve similar scalability for screens that require more complex phenotypes related to neuronal function.

## Introduction

Traditional drug screening approaches for complex brain diseases have predominantly relied on either lab adapted cell lines, primary rodent neurons, or complex *in vivo* animal models. With the advent of human induced pluripotent stem cells (hiPSCs) that capture each patient’s unique genetic elements, it is now theoretically possible to bridge the gap between the *in vitro* and *in vivo* human models to study neurological disorders in a disease-relevant manner^1^. Along with the advances in genome editing technologies such as the CRIPSR/Cas9 system^2^, many researchers around the world have already exploited hiPSCs to tackle neurodevelopmental^3^, neuropsychiatric, and neurodegenerative diseases^4^, providing novel molecular and cellular insights into these disorders. Thus, hiPSCs provide a potentially powerful tool to dissect the functional effect of genetic variants for complex disease model development and drug development^1^.

**Figure 1:**
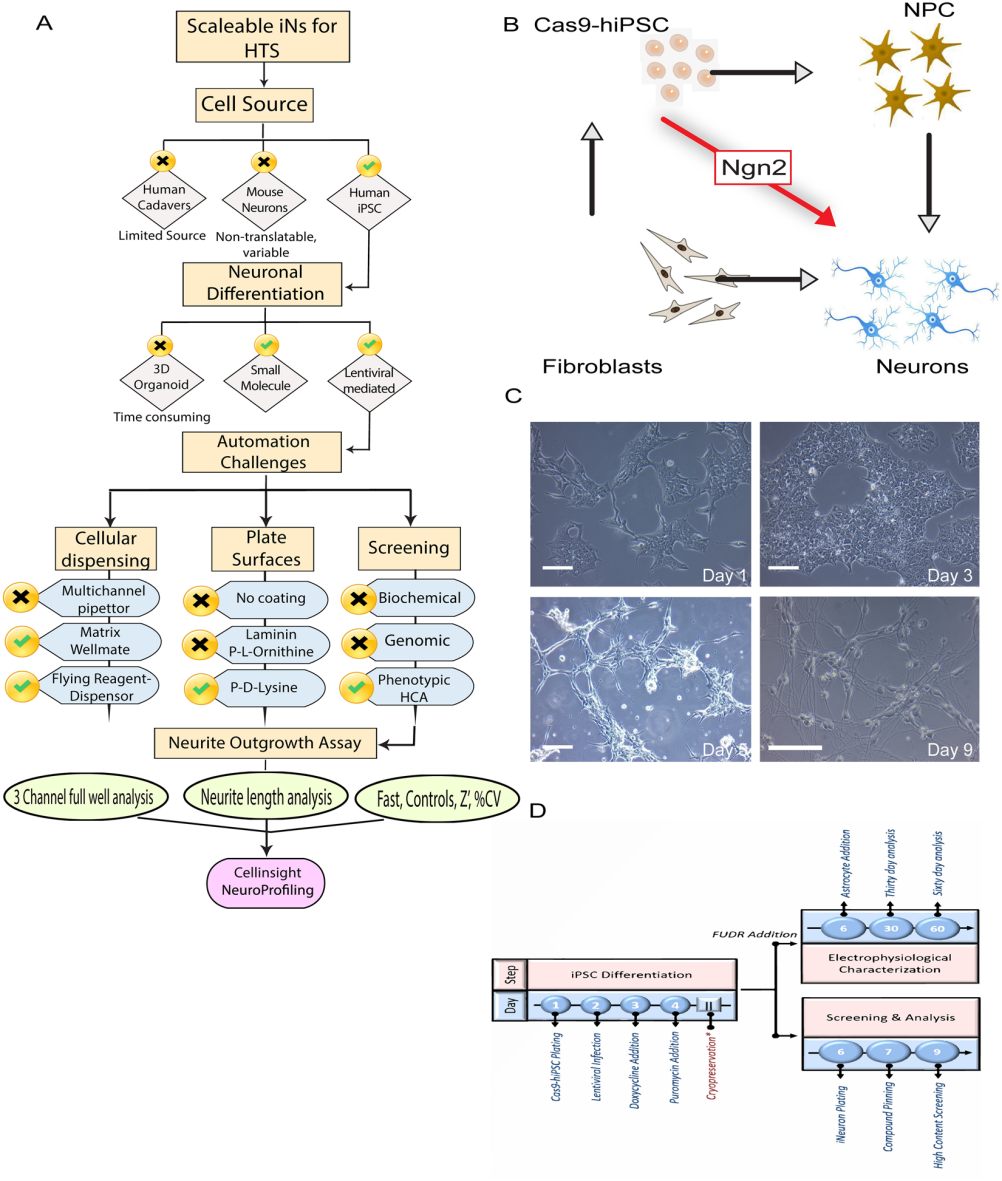
Scalability challenges and rapid generation of iNs from iPSC by transcription factor reprogramming with Ngn2. (**A**) Overview of the different scalability challenges of scaling iNs for screening. (**B**) Schematic representation of Ngn2 transcription factor-based neuronal induction starting from Cas9-hiPSC. (**C**) Representative bright-field images of hiPSC differentiation to Ngn2-induced iNs at relevant time points. Scale bar = 100 µm. (**D**) Timeline of Ngn2-induction strategy. *Represents the point where cells can be cryobanked if necessary.

Due to their ability to divide and maintain pluripotency indefinitely, hiPSCs present a promising strategy to address some of the scalability challenges while potentially mitigating the non-physiological drawbacks of using immortalized cell lines^5–7^. hiPSC-based assays allow for drug combinatorial screens^8,9^ and dose assessments^10,11^ that are exceptionally challenging to implement in existing animal models. They enable phenotypic assays and corresponding screens that more closely approximate neuropsychiatric disorders as patient-derived lines can be differentiated into functional neuronal networks within HTS compatible assay plates. Although physiologically superior to the immortal cell lines, the perceived heterogeneity and lack of cellular complexity of iNs may result in differential activity of compounds when used on end-target human cells^12,13^. As learned from parallel oncological experiments, the lack of a biomimetic microenvironment may result in an underdeveloped grasp of disease physiology leading to poorly designed *in vitro* studies and ultimately ending in ineffective clinical treatment^14–16^. Therefore, a major challenge in the field is to develop approaches to reduce heterogeneity, while at the same time increasing scalability of neuron-based assays built from hiPSCs (Fig. 1).

The availability of large numbers of post mitotic, differentiated neurons is essential for the creation of disease-relevant drug discovery assays for brain disorders. Therefore, a major roadblock to using iNs as a tool for drug discovery is to create methods that produce large quantities of differentiated neurons that can serve as the foundation for HTS-ready assays^17,18^. Currently, it remains unclear to what extent human neurons can scale in similar types of HTS-amenable phenotypic assays. Indeed, several differentiation approaches have been developed to convert fibroblast to hiPSCs, and then hiPSCs to neuroprogenitor cells (NPC) or even directly to neurons summarized in Table 1^19–22^. Three dimensional substrates including peptide hydrogels have been developed to mimic the microenvironment of iNs, but these approaches present undesirable side effects, employ multistep procedures, are not homogeneous, lack scalability, and are currently too costly for large scale screening^23,24^. Neuronal differentiation strategies can be broadly divided into small molecule-based and pro-neuronal transcription factor-based differentiation^19,21,25,26^. Although an increasing number of publications have solely focused on producing refined techniques for specific subtypes of neurons with morphological and functional uniformity, none approach the necessary scale nor are validated in a true HTS campaign^27–29^. Moreover, these other studies do not address potential limitations for screening assays that approximate physiological conditions observed in human brain disorders.

**Table 1:** A brief summary of current iN conversion strategies: commonly employed iPSC based conversion strategies are enlisted with focus on screening and challenges for scalability.

S. No	Conversion Strategy	Method	References (PMID)	Differentiation Timeline	Special Reagents	Compound Screening (Output)	HTS (384 or 1536)	Rate Limiting Steps
1	Human Fetal Brain Tissue	Tissue digestion and cellular isolation	25223359, 2224523	Post-mitotic isolated neurons are limited and not scale-able	Limited tissue source, growth factor and plate coatings	Small scale libraries	—	Source, heterogenous population
2	hiPSC → NPC → iN	Transcription factor, neural rosette formation	28246330, 25742222, 20160098, 22923789	5+ weeks total	Media, growth factors, plate coatings and	Kinase inhibitors, FDA approved library, LOPAC	384	Rosette EB formation, multistep media change and purification steps
3	hiPSC → NSC → iN	Maturation and differentiation media	24019252, 26254731	iPSC to NSC = 2–3 weeks; NSC to iN = 1–2 weeks	Patient-derived cell line	Custom library, NCE, Summit PLC	384	Multi-step, NSC differentiates into more than 1 type of neuron
4	Fibroblast → hiNSC → hiN	Vector (transcription factor), small molecule	27281220, 24379375, 26253202	4 weeks+	Patient-derived fibroblasts, growth factor and coating		384	manual colony picking, multiple purification steps
5	hiPSC → iN	Vector (transcription factor), small molecule based	23764284	2–6 weeks	Lentivirus, patient derived iPSC	RNA seq, LOPAC	384	Reagent cost, duration of screen
6	NT2 → iN	Growth factor and media change	19377856, 21331625	2 months	Commercially available	Control compounds	No	Purification steps, manual heavy, heterogenous population
7	hiPSC/ESC → EB/Organoids	Small molecule and bioreactor, embryoid body and Rosette formation, maturation media	26005811, 27934939, 29470464, 28878372	1 month+	Bioreactor, patient cell lines, growth factor cocktails	No screens reported so far	No	Non-region specific and variable, organoid production, long differentiation time, currently low throughput

In this study, we present a simplified chronological step-by-step procedure entailing the different aspects of producing and screening iNs from start to finish. This process was implemented using readily attainable and commonly accessible reagents and instrumentation. The process resulted in a robust freezer-ready, highly scalable high content screening (HCS) assay to identify compounds that cause neurotoxicity or regulate neurite outgrowth, which promises a path forward for investigators interested in identifying targets for early stage human neuron disease models. This technique can be easily adapted to other neuronal subtypes owing to its short duration in design, nine days from start to finish, and limited manipulation during screening. Future efforts will focus on improving scalability of iNs for more complex assays that measure aspects of neuronal function relevant to a range of brain disorders.

## Materials and Methods

### Cells

All products were purchased from Thermo Fisher Scientific unless otherwise mentioned. The stable human episomal Cas9 hiPSC cell line was obtained from Thermo Fisher Scientific (cat. no. A33124) and was expanded according to the manufacturer’s suggested protocol. Briefly, TC treated T175 (cat. no. 12562000) were coated with Vitronectin-N (cat. no. A14700) diluted 1:100 in DPBS (cat. no. 14190094) and incubated at 37 °C for at least 1 h prior to hiPSC plating. Subsequently, cryopreserved hiPSC cells were gently thawed for 2–3 min in a 37 °C water bath and carefully transferred to a 15 mL conical tube with complete hiPSC medium composed of Gibco^TM^ Stemflex^TM^ medium kit (cat. no. A3349401) and 1% Antibiotic-antimycotic (cat. no. 15240062). Cells were then centrifuged at 200 g (Allegra 6KR Centrifuge, Beckman Coulter, Indianapolis, IN) for 5 min after which the supernatant is carefully aspirated and hiPSC pellet is re-suspended in fresh medium and plated on VTN-coated flasks. To enhance cell viability of the cryo-recovered cells, 1% RhoK inhibitor or RevitaCell^TM^ supplement (cat. no. A2644501) was added to the complete medium for 24 h. At 70–80% confluency, cells were harvested with TrypLE Select (cat. no. 12563011) and further maintained as feeder free cells using the procedure described above.

293 FT cells (cat. no. R70007) which are used for virus production were propagated according to manufacturer’s instructions. Briefly, a cryopreserved vial was thawed in a 37 °C water bath and prewarmed in 2 mL complete medium containing DMEM (cat. no. 11965-092), 200 mM L-glutamine (cat. no. 25030-081), 10 mM MEM Non-Essential Amino Acids (cat. no. 11140-050), 100 mM MEM Sodium Pyruvate (cat. no. 11360-070), and Penicillin-Streptomycin (cat. no. 15070-063). Cells were transferred to a 15 mL conical tube and centrifuged at 200 × g, supernatant was removed, and the pellet was resuspended in fresh medium and counted using the Countess cytometer (cat. no. C10227). 3 million cells were resuspended in 10 mL complete medium and transferred to a TC treated T75 (cat. no. 13-680-65, Fisher Scientific) that was incubated overnight in a 37 °C humidified atmosphere incubator with 5% CO_2_ for cell adherence. The next day, medium was removed and replaced with complete medium containing 500 µg/mL Geneticin™ selective antibiotic (cat. no. 10131-035). Upon 75–80% confluency, cells were lifted from the flask using Trypsin-EDTA (cat. no. 25300-054) and sub-cultured or cryobanked using the Recovery^TM^ cell culture freezing medium (cat. no. 12648010) with each vial containing at least 2 million cells.

### Cryopreservation and mycoplasma testing

Upon ideal confluence estimated to be ~60–75%, hiPSCs were harvested from several flasks, pooled, counted and resuspended in an appropriate amount of ice cold primary stem cell cryopreservation medium (cat. no. A2644601) and stored at 2 million cells per mL per cryovial. The vials (cat. no. W985864, Wheaton, Milville, NJ) were labeled, barcoded (cat. no. 73314, Wheaton) and transferred to Nalgene’s Mr. Frosty Cryo 1 °C Freezing Containers (cat. no. 5100-0001) and stored at −80 °C overnight and then transferred to liquid nitrogen tank for long term storage. Each vial has a unique RFID that can be recorded by a web-based frozen sample management software (FreezerPro, Brooks Automation, Inc) for enhanced accuracy of lab operations and availability of lab information. Several vials of early passages were propagated and cryobanked in this manner. Sample vials were shipped to a third-party GMP service for mycoplasma testing using the direct culture method with DNA fluorochrome staining assay (cat. no.M-250, Bionique, Saranac Lake, NY). Samples were determined to be mycoplasma negative and the full report is appended in the supplemental images (Figure S1).

### Plasmid generation

The Ngn2 transcription factor mediated differentiation protocol uses two lentiviral constructs as described previously^21^ (i) lentivirus vector (TetO-NGN2-puro) with Addgene plasmid accession ID: 52047, and (ii) lentivirus vector (FUW-M2rtTA) with Addgene plasmid accession ID: 20342 and an optional (iii) lentivirus vector (Tet-O-FUW-EGFP) with Addgene plasmid accession ID: 30130. These Addgene plasmids (Cambridge, MA) which are publicly available were received as bacterial agar stabs and subsequently streaked on agar plates containing ampicillin (cat. no. Y2141, Teknova, Hollister, CA) to isolate single colonies that were then individually picked and used to inoculate LB broth (cat. no. 10855-001) containing 100 µg/mL ampicillin (cat. no. 11593-027) and incubated at 37 °C for 12–18 h shaking at 250RPM in a Innova 42 shaker incubator (New Brunswick Scientific, Edison, NJ). Glycerol stocks of each of the plasmids were created for future use. Plasmid DNA was isolated using mini-prep columns (cat. no. 27104, Qiagen) and purity and yield were determined using Nanodrop 2000 (cat. no. ND-2000). Individual plasmid DNA were incubated with appropriate restriction enzymes (New England Biolabs, Ipswich, MA) and NE Buffer (cat. no. B7201, NEB) for 1 h. Purple loading dye (cat. no. B7024S, NEB) was added to each sample which were loaded carefully on MiniReady Agarose Gel prepared in 1XTBE (cat. no. 101-3004, Biorad) alongside with Quick load purple 1 kb DNA ladder as a reference standard (cat. no. N0552G, NEB). The results demonstrated appropriate digest products for each plasmid as well as the maxi-prep RFLP and can be found in the supplemental data (Figure S2).

### Lentivirus production using virapower system

Thermo’s ViraPower™ Lentiviral Expression System (cat. no. K497500) was used to generate replication incompetent HIV-1 based lentivirus. On day 1, 293 FT cells were plated to be grown to 90–95% confluency and on day 2 the cells were co-transfected using Virapower packaging mix, pLenti vector (isolated from above) and Lipofectamine 2000 (cat. no. 11668-019) using OptiMEM serum-free medium. At day 3, medium was refreshed with regular growth medium and virus containing supernatants were harvested after 48–72 h post transfection. Supernatants were centrifuged at 3000 RPM for 15 min at 4 °C to pellet the debris. Finally, they were filtered with Millex HV 0.45 µm PVDF filter (cat. no. SLHVM33RS, Millipore) and stored in 1 mL cryovial aliquots at −80 °C. Virus titer was estimated based on 2-fold serial dilution across 10 points with several replicates per concentration. The GFP virus was used as a reference to predict the concentration at which 50% of the cell were infected; i.e. fluoresced green when excited at 530 nm using a fluorescence microscope.

### Generation of iNs from Cas9 hiPSC

Ngn2 transcription factor induced iNs were generated as previously described^21^. Briefly, Cas9-hiPSC cells were harvested using TrypLE Select and 2 million cells were plated on VTN-coated T75 flask on day 1. On day 2, medium was removed, and an appropriate amount of virus estimated at an MOI of 1 for both Ngn2 and rtTA was added to the cells in Stemflex medium including RevitaCell under a BSL-2 hood. After 24 h, virus was aspirated and replaced with Neurobasal medium (cat. no. 21103049) with GlutaMAX^TM^ Supplement (cat. no. 35050061), B-27^TM^ Supplement (cat. no. 17504044) and 2 µg/mL doxycycline (cat. no. 10592-13-9, Frontier Scientific) to induce TetO gene expression. On day 4 and 5, medium was refreshed with the same medium formulation and additionally 2 µg/mL puromycin was included for selection (cat. no. A1113803). Three days after lentiviral induction, iNs were harvested using Accutase (cat. no. A1110501) and plated on P-D-L coated 384 well plates (cat. no. ABE2-41201-B, Aurora Biotechnologies, Whitefish, MT) in plating medium composed of Neurobasal medium, B27/GlutaMAX Supplement, 2 µg/mL and 20 ng/mL of growth factors BDNF, GDNF and NT3 (Peprotech, Rocky Hill, NJ) or cryobanked for future experiments as follows. After Accutase treatment iNs are pelleted at 200 X g and resuspended in Synth-a-freeze cryopreservation medium (cat. no. A1254201) with each cryovial containing at least 5 million cells. The cells are stored at −80 °C in Mr. Frosty before transferring to liquid nitrogen storage the following day.

For HCS, immunostaining, and western blots, cells were analyzed 72 h after plating. For electrophysiological recording, iNs were sub-plated on P-D-L coated coverslips and medium was changed every 3–4 days until day of analysis.

### Gene expression analyses

Cas9-hiPSCs were assessed for pluripotency using the Applied Biosystem’s^TM^ TaqMan^TM^ hPSC Scorecard^TM^ Panel (cat. no. A15870) gene expression assay. Briefly, cells were harvested using TrypLE Select and RNA was isolated using the RNA isolation kit (cat. no. 74134, Qiagen) and quantified using the Nanodrop 2000. 1 µg RNA was converted to cDNA using the high capacity cDNA reverse transcription kit (cat. no. 4368814) and diluted with the TaqMan mastermix according to manufacturer’s instructions. The samples were then loaded into a pre-plated and convenient dried-down hPSC score card 96 well-plate and mRNA levels were quantified using the Step One Thermocycler in a pre-loaded software template that is available online (cat. no. 4376600). The results were then analyzed using the free hPSC scorecard cloud-based software that allows viewers to export graphs and generate reports. Please refer to the supplemental data for an example of scorecard assay layout and results.

For quantitative RT-PCR analyses of iNs and control hiPSCs, RNA was isolated using the RNA isolation kit and quantified using the Nanodrop 2000. RNA was then converted to cDNA using the high capacity cDNA reverse transcription kit. cDNA was diluted using DEPC treated water (cat. no. 46-2224) to a final concentration of 20 ng/µL per well and mRNA levels were quantified by RT-PCR assay using the Step One Plus Thermocycler. Final gene expression levels were quantified using the delta C_T_ method (Figure S3). Complete information on TaqMan assays used in the experiment can be found in Supplementary Table 1.

### Western Blot

Induced iNs at day 9 and control hiPSCs were lifted from TC treated flasks using Stem Pro Accutase and TrypLE Select, respectively. The cells were centrifuged, and pellets were homogenized in RIPA buffer (cat. no. 9806 S, Cell Signaling Tech) with Protease and Phosphatase Inhibitor Cocktails (cat. no. 11697498001 and 4906845001, Roche Diagnostics). Protein concentrations were measured with BCA (cat. no. 23225, Pierce). 5 μg of protein per sample were loaded and separated by Stain-Free SDS-PAGE on 4–15% gradient gels (cat. no. 4568086, BioRad). Following UV activation, Stain-Free gels were transferred to low fluorescence PVDF membranes (45 μm) with Trans-Blot Turbo System (cat. no. 1704272, BioRad). Membranes were probed with anti-Map2 (1:1000, 188 004, Synaptic Systems) and anti-NeuN (1:1000, cat. no. 266 004, Synaptic Systems). Chemiluminescence measurement was performed via ECL (cat. no. 32106). The original raw images are appended in Figure S4.

### Immunocytochemistry

Pluripotency of hiPSCs was further assessed by immunocytochemistry (ICC) using antibodies directed at pluripotency markers anti-SSEA-4 (1:40; cat. no. 414000) and anti-OCT 4 (1:40; cat. no. A13998). Briefly, hiPSC were plated on 384-well plates at 2000 cells/well in complete Stemflex medium and allowed to sit overnight in 37 °C incubator at 5% CO_2_. The next day, medium was removed, cells were fixed with 4% paraformaldehyde (cat. no. 252549, Sigma) prepared in DPBS for 15 min followed by incubation with primary antibody prepared in 0.1% BSA (cat. no. BP671-10). Cells were individually labeled with anti-OCT 4 (4 h incubation at 37 °C) and anti-SSEA-4 (12 h incubation at 4 °C) and washed two times with DPBS. Columns of cells were then stained with secondary conjugated antibody for 1 h and similarly after 2 washes with DPBS, cells were counterstained with HOECHST and imaged on the Cellinsight imaging reader with appropriate filters.

ICC was performed at day 9 using cryo-recovered iNs and control hiPSC and stained for dendritic marker MAP2 (anti-MAP2 1:1000; cat. no. 188 004, Synaptic Systems). For day 9 cultures, iNs were cryothawed as described before and plated on 0.1 mg/mL P-D-L (cat. no. P7280, Sigma) coated CellBind 96-well plates (cat. no. 66025-630, Corning) at 10,000 cells/well in plating medium, stained and imaged after 72 hrs. Control group hiPSCs were also thawed and plated on VTN-coated surface, stained and imaged at 72 hrs as well. At corresponding end points, spent medium was removed from wells, cells were fixed for 15 min with a 4% paraformaldehyde/sucrose solution [10 mL of 16% paraformaldehyde (cat. no. 15710, Electron Microscopy Sciences) and 1.6 g of sucrose (cat. no. S0389, Sigma) prepared in 30 mL DPBS followed by permeabilization in 0.2% Triton-X 100 (cat. no. T9284, Sigma) in DPBS]. Between every step, the cells were gently washed three times with DPBS. After initial blocking with 10% BSA for 1 h at RT (cat. no. AK1391-000, AKRON), the cells were incubated with primary antibody prepared in 0.1% BSA. Cells were individually labeled with anti-MAP2 (1 h incubation at RT). Cells were then stained with secondary conjugated antibody Alexa 488 (1:500; cat. no. A11073) for 1 h at RT and then counterstained with DAPI stain (1:1000; cat. no. 62248) for 5 min at RT and Sodium Azide (cat. no. 58032, Sigma) 0.01% w/v in DPBS was then added as a preservative. The cells were immediately imaged on the IN-Cell Analyzer 6000 laser-based confocal imaging platform with appropriate filters. The entire list of antibodies used in the immunocytochemistry experiment is enlisted in Supplemental Table 2.

### Electrophysiology

**Figure 2:**
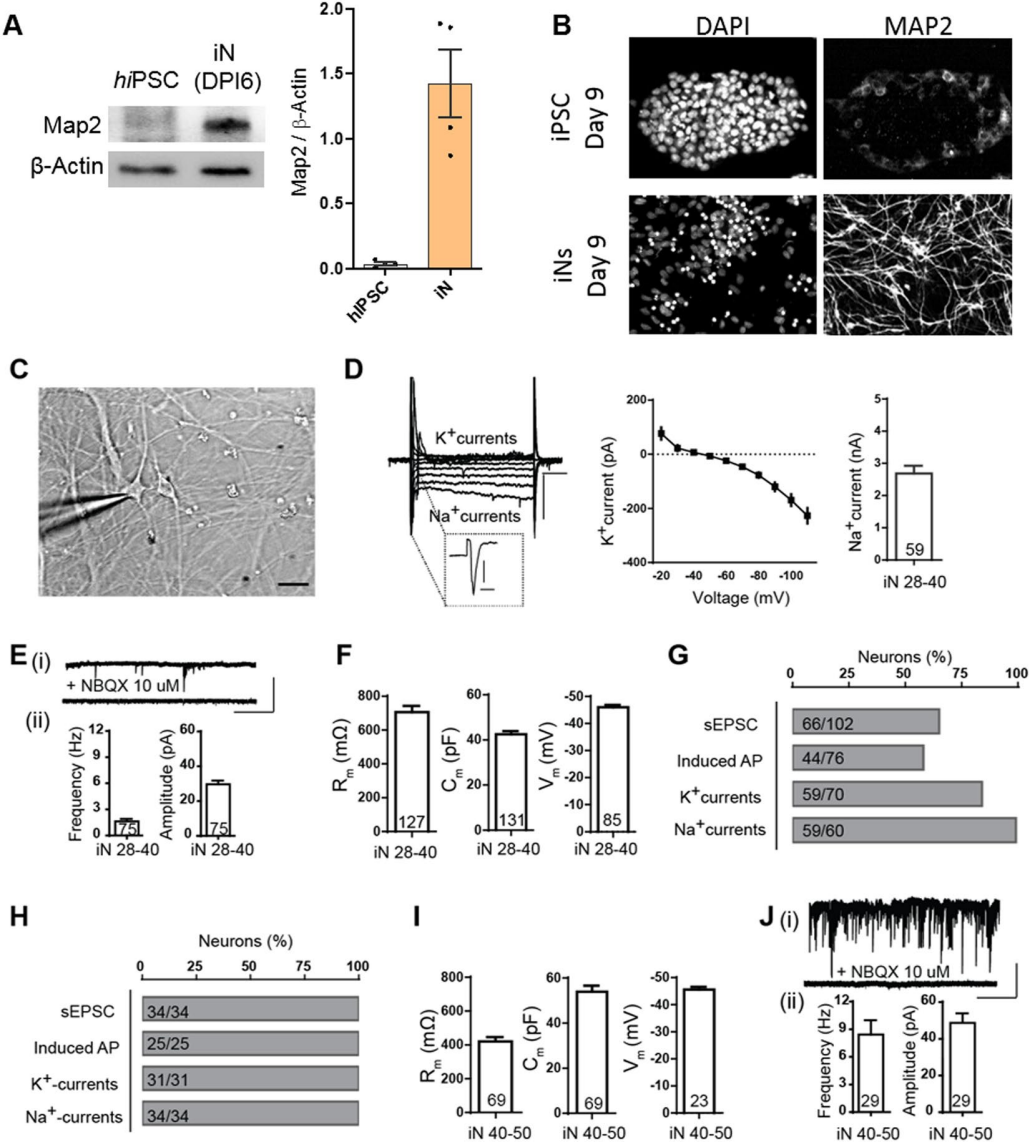
Functional Characterization of Ngn2 generated iNs. (**A**) Immunoblot of cellular homogenates from hiPSC or induced neurons at day 6 post induction (DPI6) which is the same as day 9 in culture. Data points on plot represent biological replicates. Gel membranes were cut before probing for mature neuronal markers Anti-MAP2 Ab (280 kDa) with loading control Anti-β actin Ab (42 kDa). The samples are derived from the same experiment and the gels were processed in parallel, the images pertinent to the appropriate lanes have been cropped and assembled for clarity. (**B**) Representative immunocytochemical staining images of iNs showing positive for dendritic marker MAP2, at day 9. In all images, nuclei were labeled by DAPI. (**C**) DIC image of a patched iN at day 30 (scale bar 20 µm). (**D**) Representative traces of whole-cell voltage-clamp of Na+ currents (inset, scale bars 1 nA, 5 ms) and K+ currents (scale bars 200 pA, 100 ms), I/V quantification of K^+^ currents (middle) and quantification of Na+ currents in recorded in iNs 28–40 DIV (right). (**E**) Example traces of sEPSCs (i), lower trace displays block of sEPSCs by the AMPA receptor antagonist NBQX (scale bars, 100 pA, 5 s). (ii) Graphs of the frequency (left) and amplitude (right) of sEPSCs in iNs 28–40 DIV. (**F**) Input resistance (left), capacitance (middle) and membrane potential (right) of iN 28–40 DIV. (**G**) Percentages of successful observations of sEPSCs, current-induced AP, K+ currents and Na+ currents in iNs 28–40 DIV (number of successful observation in iNs/total iNs patched is indicated in bars). (**H**) Percentages of successful observations of sEPSCs, current-induced AP, K+ currents and Na+ currents in iNs 40–50 DIV (number of successful observation in iNs/total iNs patched is indicated in bars). (**I**) Input resistance (left), capacitance (middle) and membrane potential (right) of iNs 40–50 DIV. (**J**) Example traces of sEPSCs (i), lower trace displays block of sEPSCs by the AMPA receptor antagonist NBQX (scale bars, 100 pA, 5 s) (ii) Graphs of the frequency (left) and amplitude (right) of sEPSC in iNs 28–40 DIV. Bars represent means ± SEM. Values in bars indicate the number of iNs.

We characterized iN function by culturing hiPSCs expressing Ngn2 for up to 50 DIV and then performing whole-cell patch-clamp recordings (Fig. 2). For these studies, the iNs were cryo-recovered and simultaneously co-cultured with cryo-recovered primary rat astrocytes (seeded at 20,000 iNs + 10,000 astrocytes per well) in 24-well plate on 15 mm coverslips. The co-cultures were maintained in plating medium and additionally supplemented with 10 µg/mL FUDR (cat. no. F0503, Sigma), indicating that all cells were postmitotic. Briefly, intrinsic electrical properties and synaptic activity were recorded at room temperature in voltage-clamp configuration (cells were held at −60 mV with a Multiclamp 700B amplifier, Molecular Devices) and switched to current-clamp mode to record firing properties. Pipettes pulled from borosilicate glass capillary tubes (cat. no. G85150T-4, Warner Instruments) using a P-97 pipette puller (Sutter Instrument) were filled with the following intracellular solution (in mM): 123 K-gluconate,10 KCl, 1 MgCl_2_, 10 HEPES-KOH, 1 EGTA, 0.1 CaCl_2_, 1 K_2_-ATP, 0.2 Na_4_-GTP and 4 glucose (pH adjusted to 7.4 with KOH). In some cases, the intracellular solution contained the following (in mM): 120 CsCl, 5 NaCl, 1 MgCl_2_, 10 HEPES-NaOH, 10 EGTA, 3 Mg-ATP, 0.3 Na_4_-GTP (pH adjusted to 7.4 with NaOH). Resistance of the pipettes filled with the intracellular solution was between 3.5–5 mΩ. The bath solution contained (in mM): 140 NaCl, 5 KCl, 2 CaCl_2_, 2 MgCl_2_, 10 HEPES-NaOH and 10 Glucose (pH to 7.5 adjusted with NaOH). Series resistance was monitored without compensation with 5 mV depolarizing steps (200 ms) induced every 60 s to ensure sufficient electrical access to the cell. Action potentials were elicited by injecting currents from 0 to 180 pA in 20 pA steps (700 ms per step).

Data were sampled at 10 kHz and analyzed offline using Clampfit (Molecular Devices). Single peak sEPSCs were detected using a semiautomated sliding template detection procedure in which the template was generated by averaging multiple spontaneous currents. Each detected event was visually inspected and discarded if the amplitude was <7 pA.

### Karyotype analysis

Cas9-hiPSCs and differentiated iNs from two different lots were assessed for any chromosomal aberrations using the Karyostat^TM^ assay. Briefly, gDNA from cells was prepared according to the genomic DNA purification kit (cat. no. K0512) and quantified using the Qubit^TM^ dsDNA BR assay (cat. no. Q32850). 250 ng of gDNA was processed and hybridized onto a Genechip® array for Karyostat^TM^ according to the manual.

### Compound library

In the present proof-of-concept pilot screen, DMSO and control drug concentration response curve (CRC) plates were executed at the beginning sequence followed by the LOPAC^1280^ collection (Sigma Aldrich, St. Louis, MO). Compounds were pinned from source plates as 2.5 mM solutions in DMSO. The final nominal compound concentration in the assay was 8.5 µM and the final DMSO concentration was 0.25%.

### A 384-well assay protocol

**Figure 3:**
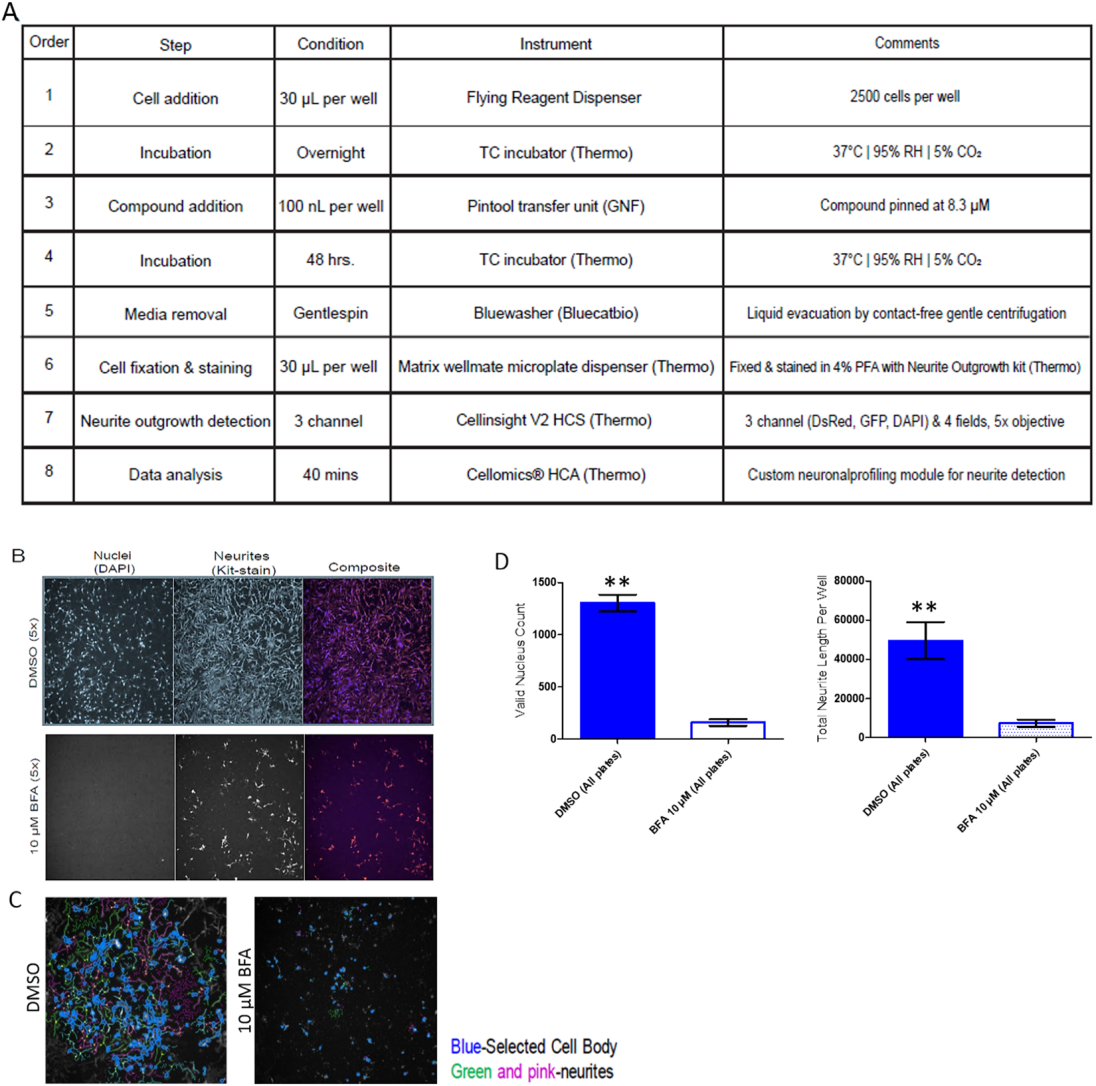
Development and validation of HCS assay to screen for neurotoxic compounds in iNs. (**A**) Stepwise protocol for the 384-well plate neurotoxicity assay starting from hiPSC differentiation to HCS data analysis. (**B**) Representative fluorescent high content images of iNs with Channel 1 corresponding to nuclei (DAPI) and Channel 2 for neurite outgrowth (DsRed) and a composite merged image of the 2 channels. The groups selected corresponded to low (DMSO treated) and high control (10 µM Brefeldin A-BFA treated) wells. (**C**) Representative fluorescent high content image processing of same images where neurites were traced according to predefined optimized parameters under the neuronal profiling module of the CellInsight software. Each image corresponds to 1 field of view and each well has 4 fields of views using the 5X objective. (**D**) Automated quantification of valid nucleus count, neurite count, neurite length from iNs treated with control compounds DMSO and 10 µM BFA. Data are from 4 independent experiments, total n = 12 biological replicates per experiment; values are means ± stdev. **p < 0.0001.

A detailed stepwise protocol is presented in Fig. 3A. Briefly, 48 h after purocmycin selection, 2500 iNs in 30 µL of plating medium were seeded in black clear bottom P-D-Lysine coated, square IQ-EB 384 well plate (cat. no. ABE241201-B-PDL) at 2500 cells per well using Flying Reagent Dispenser (FRD, Aurora Biosciences). After an overnight incubation (~16 h) at 37 °C, 95% relative humidity (RH), and 5% CO_2_, cells were treated with 100 nL test compounds or DMSO using a pintool transfer unit (Kalypsys/GNF). Plates were further incubated for 2 days at 37 °C, 95% RH, and 5% CO_2_. The differentiated iNs were then fixed with 4% PFA and stained with Neurite Outgrowth Kit (cat. A15001, Thermo) and Hoechst (cat. H3570, Thermo) for nuclei staining and each well was imaged on a high-content reader as described below.

### High content image acquisition and analysis

Following treatment and incubation as described above, medium was removed using a BlueWasher (BlueCat Bio, MA) using the gentle spin mode and cells were stained by Neurite Outgrowth Staining kit using manufacturer’s instructions (cat. no. A15001). Using a Matrix WellMate 30 µL of 4% PFA containing HOECHST was added to each well and incubated at 37 °C for 20 min to fix and stain the cells. Wells were voided of liquid using BlueWasher, the plates were washed with DPBS followed by addition of 30 µL per well background suppression dye provided in the Neurite Outgrowth Staining kit. The plates were then imaged under 2 channels under DAPI and dsRed (Fig. 3B).

A protocol was developed to read the plates using the CellInsight high-content reader (Thermo Fisher Scientific, Pittsburgh, PA) using a 5x objective collecting 4 fields of view per well. An illustrated description of the image analysis and labeling schema is presented in Fig. 3C. Briefly, the CellInsight’s Neuronal profiling assay algorithm was optimized for the screen. Primary objection identification was designated to identify nuclear staining by HOECHST in channel 1 (386 nm at 0.06 s exposure time). Neurites were labeled with the cell membrane stain identified by acquiring the dsRed channel as channel 2 (546 nm, 0.053 s exposure). Primary objects were identified using the isodata threshold method. Valid cell bodies are identified using isodata method requiring a minimum cell body nucleus and were segmented using nuclei and an optimized demarcation parameter. Neurites were identified by the binomial method with a value of 3 and a neurite identification marker of −0.98 that is further refined by a direction length and point resolution. Based on optimization, an intermediate gap tolerance of 10 was set to allow for detection of neurites without seeds (which are defined as a cell body) thereby assisting with aggressive tracing.

Using the Z′ ranking tool of the HC reader, 3 important output features were exported for each plate (i) valid nucleus count that describes the viable neurons in the acquired fields of view (ii) total neurite count per well that accounts for selected neurites in the entire well (iii) total neuronal length feature that provides the sum total length of individual neurites in artificial units in all the 4 fields of view predefined by the neurite identification marker.

### Data management and HTS data analysis

Upon plate-based data acquisition using the Cellinsight exported data files, i.e. associated to the three parameters above, were uploaded to Scripps’ institutional database (Symyx Assay Explorer), where plate statistics (Z, Z′, S/B) were automatically calculated. Compound heredity was automatically retrieved from Scripps’ compound management database (Plate Manager, MDL), and percent neurotoxicity was calculated for each compound using the following equation:

$${\rm{ \% }}neurotoxicity=100\times \frac{Testwell-Median\,Low\,Controls}{Median\,High\,Controls-Median\,Low\,Controls}$$

where Testwell refers to compound wells and High and Low Control wells represent wells treated with 10 μM Brefeldin-A and DMSO, respectively. Modeling the normalized data on a standard distribution for all test wells we found a response of 40% to 100% to occur for those samples that were one standard deviation above the mean. These compounds were designated as hits.

### Statistical analysis and graphs

All scatter-plots and graphs included in the article have been made with GraphPad Prism (GraphPad Software, La Jolla, CA). Statistical analyses were performed using unpaired Student’s t-test (GraphPad) between the test and the control groups. The statistical significance threshold was set at 0.05 for all tests (with p < 0.05).

## Results

### Characterization and Scale-up of Ngn2-transduced hiPSCs:  *Generating Large Batches ofHuman Glutamatergic Neurons Suitable for Screening*

High-throughput screening campaigns require that iNs are produced in a scalable, homogeneous, reproducible and affordable manner. Thus, key parameters to achieve HTS scaling of iNs using lentiviral mediated Ngn-2 expression are: (i) a high quality pluripotent hiPSC line, (ii) determination of lentiviral titer and infectivity, and (iii) employing robust assay methods. Our goal was to create a workflow that was generally accessible to the academic research community. We evaluated several protocols for producing iNs from hiPSCs and selected the method from Zhang *et al*.^21^. We chose this method because it appeared to be the most efficient protocol for creating iNs, which results in functional glutamatergic neurons after only a few weeks in culture. Importantly, we optimized this existing iN differentiation protocol^19,21^ for use in screening environments using materials and reagents from commercial vendors **(**Fig. 1A–D**)**.

We chose the Cas9 eipsiomal hiPSC line, which stably expressed CAS9, because it would in theory would facilitate CRISPR-based genetic screens in human neurons. Additionally, this cell line is also guaranteed pluripotency for up to 50 passages, a key requirement for large batch differentiation and screening. As a first step, the hiPSC Taqman Scorecard kit was used to verify trilineage differentiation potential by gene expression the commercially-obtained hiPSC line. Scorecard assay provides four sections: overall summary, expression pattern plot, box plot of C_t_ values, and, correlation plot. For the hiPSC used in the study, the overall summary was (+) for self-renewal (−) for the three germ layers thus confirming pluripotency. From the expression plot, there is a 2 to 10-fold upregulation of gene expression for NANOG, POU5F1, SOX2 and TRIM22 (self-renewal); MAP2 (ectoderm) and GDF3 (mesoderm). There was a 10 to 100-fold upregulation in CXCL5 (self-renewal) and SDC2 (mesoderm) and fc > 100 upregulation in NR5A2 (mesoderm). All other genes were either undetectable or downregulated with respect to reference data set of hiPSC. A complete scorecard data analysis report can be found in the supplementary information (Figure S5A). Secondly, we characterized pluripotency by standard immunocytochemical staining assay. The cell line used in the study demonstrated homogenous and strong staining of pluripotency markers OCT4 and SSEA4 (Figure S5B).

Second, all plasmids were confirmed by RFLP method demonstrating appropriate base pair cuts with the restriction enzymes listed in table (Figure S2). These plasmids were then scaled and utilized for lentivirus production as described above. Since it is a replication-incompetent single round of infection virus particle system, (i.e. co-transduction of Ngn2 and rTTa), producing high titer infectious lentivirus is critical for scaling up hiPSC induction of neurons. Using the procedures outlined with the methods section, one individual can easily produce up to 60 million iNs for cryopreservation in one batch over the course of 9 days affording us enough to test 24 K wells (using the 384w format). To put this in perspective, we used 20 million post-mitotic iNs to screen the LOPAC 1280 collection in triplicate (technical replicates). Using the combination of lentiviral induction and cryobanking method, it would be straightforward to freeze down tens of millions of cells, which would allow for a full-scale compound library screen on the order of hundreds of thousands of wells.

An important aspect of our work was to confirm that cells produced from the cryobanking method had characteristics consistent with Ngn2 iNs. First, we karyotyped cryobanked iNs and control Cas9 iPSCs to look for any chromosomal aberrations that may have been introduced during generation on the iNs (Figure S6). This assay allows for a digital visualization of chromosomal aberrations with a resolution similar to g-banding karyotyping. This step was critical because the cell line of choice stably expresses Cas9, which could in theory cause chromosomal alterations during the production process. The size of structural aberrations that can be detected is >2 Mb for chromosomal gains and >1 Mb for chromosomal losses. The KaryoStat™ array is optimized for balanced whole-genome coverage with a low-resolution DNA copy number analysis, and enables the detection of aneuploidies, submicroscopic aberrations, and mosaic events. Analysis of the iNs and Cas9-hiPSC revealed a copy number (CN) of 2 across all chromosomes (except for the Y-chromosome which was not detected), for all samples. These results indicate a normal female karyotype in both the Cas9 iPSC and the iNs, thus confirming no chromosomal aberrations were introduced during the generation of iNs.

We next measured neuron-enriched protein expression and cellular morphology in puromycin-selected cells expressing Ngn2. Immunoblotting revealed that puromycin-selected cells had robust MAP2 expression a week after induction, whereas MAP2 signal was not detectable in hiPSCs (Fig. 2A). Moreover, immunocytochemistry studies revealed that Ngn2-expressing cells were highly differentiated compared to hiPSCs, with iNs having dendrite-like extensions enriched with MAP2, and these structures were absent in hiPSCs (Fig. 2B).

Third, we patch-clamped Ngn-induced, freezer-ready iNs to confirm that these cells matured into synaptically connected and spontaneously active glutamatergic neurons. To do this, we co-cultured iNs with rodent astrocytes, a requirement for Ngn2-induced hiPSCs to produce functional synapses^19^. We prepared freezer-ready rat primary astrocytes and co-cultured these cells with freezer-ready iNs as part of a streamlined protocol to produce synaptically active and functional iNs (Fig. 2C). Similar to previously published reports, sodium and potassium currents were observed within ~3–4 weeks (Fig. 2D). We also observed spontaneous release of glutamate, indicating that these neurons were synaptically connected and firing action potentials (Fig. 2E). However, iNs at this stage of development were heterogenous. As a population, membrane resistance was relatively high and capacitance relatively low, indicative of electrically non-complex cells (Fig. 2F). Moreover, many cells were devoid of neuronal characteristics, such as action potentials and synaptic activity (Fig. 2G). However, by 50 days *in vitro*, neurons were much more homogenous (Fig. 2H). iNs exhibited neuronal characteristics indicative of more mature cells, such as lower membrane resistance and higher capacitance (Fig. 2I). Furthermore, all cells recorded at this developmental stage exhibited spontaneous glutamatergic activity (Fig. 2J).

### High content assay to screen for neurotoxic compounds

We were confident that the freezer-ready iN production protocol produced cells that developed into well-connected and spontaneously active neurons. Thus, we next sought to use these cells to develop a simple phenotypic and HTS-scalable assay that is also generally relevant to brain disease. Neurite outgrowth is a fundamental neuronal process that is required for the formation of neural networks. Neurite outgrowth is potently regulated by neurotoxic compounds. To date, predominantly all the neurotoxic compound screening has been conducted in either less relevant lab-adapted immortalized cell lines such as PC-12 or PDX derived glioblastoma^30^ or rodent primary neurons that cannot replicate the physiology of the response in humans. Since the development of human-based *in vitro* models for neurotoxic screens is still relatively new, we wanted to validate our iNs by developing a 384-well format HCS assay to detect neurotoxicity in human neurons followed by testing it on thousands of compounds (Fig. 3A). To perform the study in a controlled fashion, we compared neurite outgrowth of the iNs in the sample wells with that of the Brefeldin A compound treated control wells. Brefeldin A was implemented because it is a well-known inhibitor of protein transport which ultimately served to limit neurite outgrowth and behaved as such in our experiments that served as a key feature in the detection algorithm^31^.

Several rounds of optimization were performed evaluating parameters such as (i) cell seeding density and length of incubation (ii) using cell dispenser compared to hand plating (iii) preliminary assessment of several plate types in terms of functional plate coating, evaporation and humidity consideration (iv) compatibility with dispensers and readers (v) fresh vs. cryo-thawed neurons in terms of neuronal output data all of which culminated in the conditions used to screen the pilot initiative (Supplemental Table 3).

**Figure 4:**
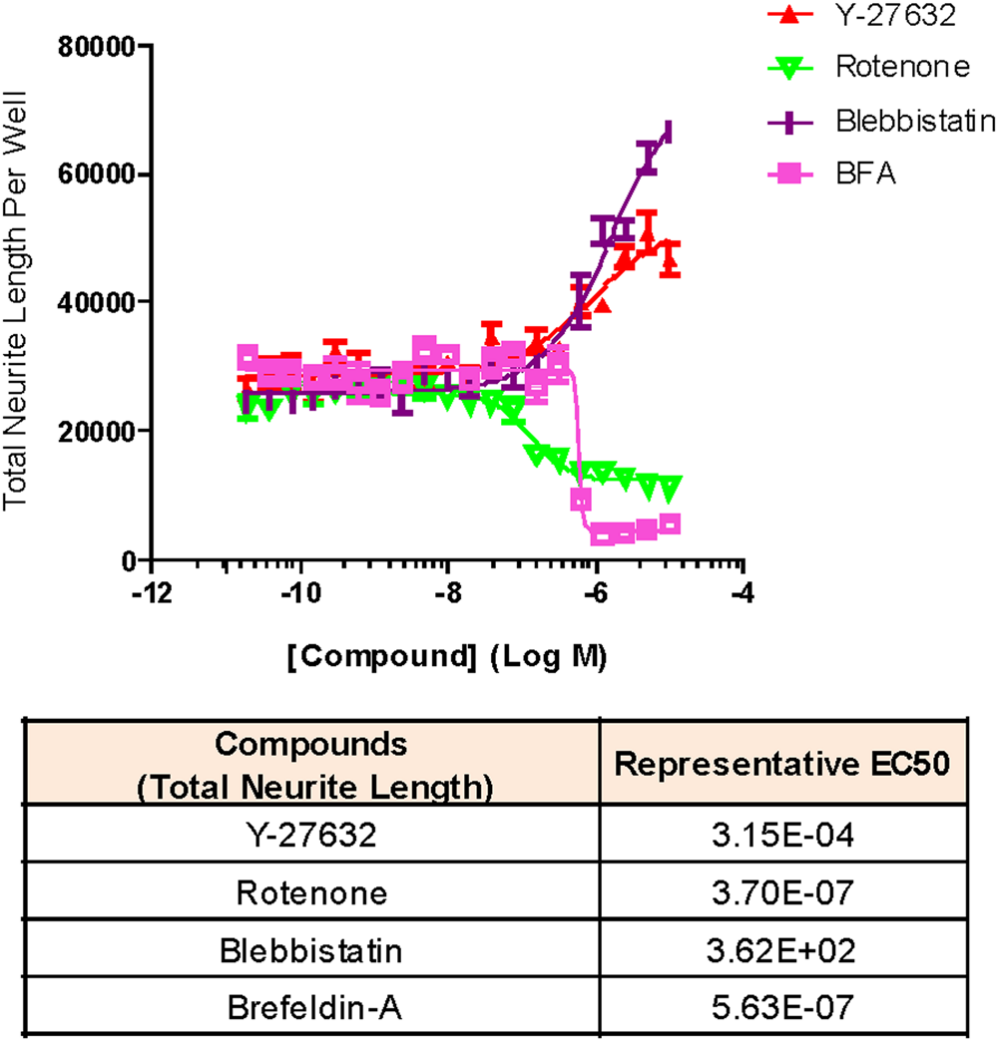
Control Compounds for Neurotoxicity assay using HCA. Representative CRCs of four control compounds (Y-27632, rotenone, blebbistatin, brefeldin-A) analyzed for their effect on total neurite length. Each curve represents mean and SEM of 4 biological replicates. Representative EC50 values for total neurite length included as a table below; Brefeldin-A average EC50 = −5.6 ± −5.5 Molar, (n = 3 experiments, technical replicates).

Assay reproducibility was assessed at three levels: 1) well-to-well, 2) plate-to-plate, and 3) day-to-day. As a first check, the valid nucleus count measure across the plates was correlated with intraplate reproducibility, which we assessed via matching coefficient of variability between the plates to ensure they correlated with each other. Day-to-day reproducibility was derived from all three parameters (valid nucleus count, neurite length per well, neurite count per well) that served as a diagnostic summary of neuronal health and thereby increased our confidence in the method. For each HTS run, several sentinel wells (i.e. those that were treated with vehicle only) were randomly chosen for morphological assessment at high magnification (20x). This internal quality control metric is essential for successful implementation of the assay using HTS-level automation.Optimization of assay conditions was done to adjust parameters that afforded the most robust iN screening system which determined the best seeding density to be 2500 cells/well. Neuronal health was assessed from total neurite count, total neurite length, and, valid nucleus count. Treatment with compounds such as Brefeldin-A and rotenone which are known to ablate neurite outgrowth^32^ reduced total neurite length and total nucleus count^33^; and treatment with compounds such as Y-27632 and Blebbistatin^33^ enhanced total neurite length in a dose dependent manner, which confirms the assay specificity and sensitivity (Fig. 4). The average Z′ factor, a measure of the assay response window^34^, was measured to be >0.50, thus affirming the robustness of the HCS assay.

### Identification and validation of neurotoxic compounds via HCS

**Figure 5:**
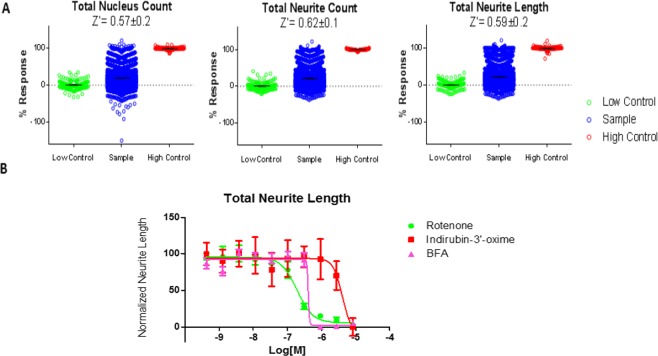
384-well Neurotoxicity pilot screen results using LOPAC. (**A**) Scatterplots of the data generated using automated quantification of neurite outgrowth detection parameters from iNs tested against the LOPAC at 8.3 µM. The library was distributed to 4 384-well compound plates; samples were run in triplicate with each plate containing 24 control wells (12 DMSO green points and 12 BFA red points). Results are shown as the % response. Hits were chosen based on their ability to achieve >50% activity in the total neurite count and total neurite length detection parameters. (**B**) CRCs of Rotenone, indirubin-3′-oxime, and BFA, tested in the iNs models of each where the bar represents the mean and SEM in triplicate. Triplicate data denotes technical replicates. **p < 0.05.

To validate the scalability and effectiveness of the HCS assay described above, we screened iNs using the LOPAC (Library of Pharmacologically Active Compounds) collection to identify neurotoxic compounds. LOPAC contains 1,280 bioactive small molecules, including inhibitors, receptor ligands, marketed drugs, and pharmaceutically relevant structures, that affect most signaling pathways and cover major drug target classes. The LOPAC collection was tested as single dose in triplicate and again Z’ scores were calculated based on low control (DMSO) and high controls (10 µM BFA). Results of the pilot screens are summarized in Fig. 5A. Activity of the compounds was determined for each of the neuronal health parameters. Compounds that significantly changed neurite outgrowth, i.e. those with activity greater than the cut-off described above, in neurite total length and total neurite count, were defined as hits. This identified 14 compounds that proceeded to a concentration response assay.

These compounds were re-ordered as fresh powders and prepared as serial dilutions and tested in triplicate, starting from ~8.3 µM nominal concentration, generating data using the same output parameters as aforementioned modality. Of the 14 compounds tested, only one compound exhibited a statistically significant concentration response, indirubin, which is a known CDK1 inhibitor (p < 0.05) (Fig. 5B). During each run we included a panel of control compounds previously known to elicit an inhibitory or increasing effect on neurite outgrowth including the Rho Kinase inhibitor Y-27632, Blebbistatin, Rotenone and Brefeldin A (Fig. 4). This data when combined with the outcome of the LOPAC pilot clearly demonstrates that neurotoxic compounds can be promptly identified by HCS assay using induced human neurons. The measurable effect of Y-27632 and Blebbistatin also demonstrated the potential for this approach to find molecules that enhance neurite outgrowth.

### Addressing scientific rigor of ins for screening assays

**Figure 6:**
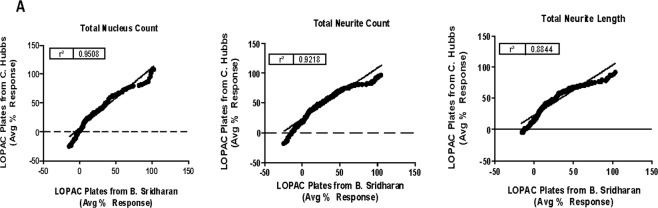
Addressing scientific rigor between different users. (**A**) Correlation plots of the activity found in the LOPAC pilot assays when testing the method in two separate labs.

The biggest roadblock to using iPSC derived iNs for drug screening, is the high degree of variability generated while scaling the clones for HTS. Here, we overcame this problem by conducting two experiments. First, we transferred the assay to an alternate lab to ensure they were able to recapitulate the same results observed for the LOPAC pilot. The data was interpreted independently using the same detection algorithm. A correlation of the activity of all compounds tested yielded an r^2^ value that was >0.88, indicating that the scalability and reproducibility of our method led to very little variation in a technique-dependent and user-independent fashion (Fig. 6).

Secondly, we compared the responses of freshly generated iNs and cryobanked iNs to compounds known to impact neurotoxicity and neurite length. We did so by measuring these parameters using the HCA and testing them simultaneously under the same conditions, on the same day, for the duration of the experiment including image acquisition and data analysis. The correlation of the data resulted in an average r^2^ value >0.42 for each of the output parameters (neurite length and valid nuclei count), demonstrating that the performance of cryopreserved cells was reasonable and can continue to be used for forthcoming assays. The hits between the LOPAC pilot tests recapitulated further increasing our confidence in the system (Figure S7).

## Discussion

The goal of this current study was to develop a method for human neuron assay development that was scalable, reliable and economical. To do this, we evaluated the performance of an iN-based, HTS-ready neurite outgrowth assay. Our approach was to bulk produce human-derived glutamatergic neurons by employing readily available reagents that support the viral transduction of a hiPSC line with the Ngn2 transcription factor^19^. We subsequently optimized this method to enable cryobanking of very large batches of iNs, a protocol capable of generating tens of millions of differentiated cells per batch. We next developed a robust phenotypic neurotoxicity assay protocol to validate cryo-banked iNs against newly differentiated iNs. This test was performed through a “side-by-side” comparison where all iNs originated from the same iPSC production run. We found that performance of the cryo-banked iNs was similar to that of freshly prepared iNs. This proof of principle pilot screen demonstrated that Cryo-iNs are sufficiently reproducible and scalable when used in HTS-ready phenotypic screening assays. Several recent studies have shown that cryobanked iNs can be used for phenotypic assays^35,36^, though to our knowledge, this is the first time that cryobanked iNs have been used successfully in a HCS effort of this magnitude. Moreover, while cryobanked iNs can be obtained commercially, these sources are severely limiting for assay development and drug discovery. Therefore, our procedure for scaling iNs unlocks the ability of a laboratory to screen custom-developed hiPSC lines, such as those derived from patients.

Using the Ngn2 protocol published by Zhang, we typically produced ~20 million iNs and a cost of $3,300, which equates to $165 per 384 w plate. Note that the rate of conversion of iPSC to iNs is heavily reliant on the Multiplicity of Infection (MOI) and the titer of the lentiviruses used. Using a MOI of 1–2, and titer of 10^8^ TU/mL, a starting cell number of 25 million iPSCs was used to produced 20 million iNs after puromycin selection. These costs do not include labor. It is possible that other approaches could be more cost-effective.

One of the crucial challenges for neuronal-HTS is addressing the heterogeneous nature of these cellular populations. Heterogeneity within cellular populations leads to assays with high intrinsic variance. Assays with high variance require large effect sizes and multiple replicates, which increases screening costs and lowers scalability. This was the rationale behind the decision to create a stable and reproducible assay using off-the-shelf reagents, including a commercially available iPSC line that stably expresses Cas9 protein. The protocol yielded acceptable assay statistics and demonstrated that iNs can now be produced and screened in high throughput settings. The assay performance might still be enhanced by opting for more expensive plate-coatings, multi-step antibody staining, and longer time points, but this pilot study is a proof-of-concept for investigators interested in human neuron early-screening and sets the stage for enhanced development. Future efforts are focusing on CRISPR-induced disease model development and advanced miniaturization to the 1,536 well-plate formats. The assay presented here represents a key stepping stone to encourage scientists to pursue human iN-based approaches for HTS on other disease models. Finally, in addition to screening neurotoxic compounds, the assay may also be used to uncover neuroprotective compounds when deployed over larger LentiArray CRISPR or siRNA libraries.

Our group has recently shown that rodent primary neurons can be used to develop HTS-compatible phenotypic assays capable of reporting dynamic changes in synaptic connectivity^37^. This assay was used in a true HTS setting to screen in excess of eighty thousand wells for novel regulators of *de novo* synaptogenesis^37^. With the successful implementation of human iNs into HTS phenotypic screening reported here, it is worth asking if iNs can replace primary rodent neurons as the principle cell-type for disease-relevant phenotypic screening assays. At this point, the choice of which neuronal source to choose is most dependent upon the desired scale of the screen and the type of phenotype to be measured. While hiPSC-derived iNs have obvious advantages related to direct disease modeling, the current generation of iN differentiation protocols suffer from limitations that greatly impact scalability for creating assays with more complex, and possibly more disease relevant, phenotypes. Phenotypic assays that require any feature of a mature network, such as synapse formation, synapse function, or neuronal activity, requires that Ngn2 iNs be co-cultured with glial cells. This co-culturing step increases the complexity of the assay and may lead to unacceptably high variance. Moreover, our studies indicate that neurons remain immature beyond one month in culture (Fig. 2). Considering that iNs require feeding every two days, this level of maintenance (i.e. thrice weekly feedings for several weeks) would greatly restrict the scale of a screen because of the need to repeatedly feed the cells. In contrast, rodent primary neurons do not require extra co-culture steps because they form active synapse in the absence of added glia. Furthermore, rodent primary cells begin to form synapses within the first week in culture^38^. Thus, functional endpoints in neurons can be achieved in a rodent model in the first week after plating. These features enable the design and planning of much more ambitious screens, albeit directed at non-human models. Indeed, rodent neurons can form functional synapses within the highest-density plates, including 1536 w format, within one week in culture^37^. Thus, for ambitious screens, where tens-of-thousands of compounds are used to find modifiers of a functional phenotypic, rodent neurons may be a more appropriate choice. In this context, hiPSC-derived iNs could be used to develop a secondary validation assay limited to a few thousand wells. However, for lower throughput or focused library screens of a functional neuronal endpoint, or for HTS campaigns for a simple assay, such as neurite outgrowth, iNs may be a more appropriate model. Indeed, future efforts will focus on improving the features of iNs to increase their scalability within HTS environments, particularly for phenotypes that report neuronal function.

## Supplementary information

Dataset
