# Supplementary material for: A Simple Procedure for Creating Scalable Phenotypic Screening Assays in Human Neurons
Source: Sci Rep. 2019 Jun 21;9:9000. doi: 10.1038/s41598-019-45265-1 (PMC6588600; doi:10.1038/s41598-019-45265-1)
Supplement: Supplementary file 1 — Dataset [file 41598_2019_45265_MOESM1_ESM.pdf]

A Simple Procedure for Creating Scalable Phenotypic Screening Assays in Human Neurons

BanuPriya Sridharan[1]; Christopher Hubbs[2]; Nerea Llamosas[2]; Murat Kilinc[3]; Fakhar U. Singhera[1]; Erik Willems[4]; David R. Piper[4]; Louis Scampavia[1]; Gavin Rumbaugh[1,2,3ɣ] and Timothy P. Spicer[1*]

[1] *The Scripps Research Molecular Screening Center, Department of Molecular Medicine, Scripps Research, Jupiter, Florida 33458, USA*

[2]*Department of Neuroscience, Scripps Research, Jupiter, Florida 33458, USA*

[3]*Graduate School of Chemical and Biological Sciences, Scripps Research, Jupiter, Florida 33458, USA*

[4]*Cell Biology, Thermo Fisher Scientific, Carlsbad, California 92008, USA*

[ɣ]Co-communicated with Gavin Rumbaugh

Corresponding Author:

Timothy P. Spicer
Scripps Florida
Department of Molecular Medicine
130 Scripps Way #1A1
Jupiter, FL 33458
(561) 596-2418
E-mail: spicert@scripps.edu

Document ID #: DCF9002I
Title: **Mycoplasma Test Final Report**
Effective Date: 03/24/17
Edition #: 07

BTL Sample ID #: **90903** BTL Test/Cat. No: **M-250** Date Rec'd: **03/30/17**
Multimedia Direct Culture with Indicator Cell Culture Assay

Client Name/Address:

BanuPriya Sridharan
The Scripps Research Institute, Florida

Test Article:
Human Cas9 IPSC; P6 Lead ID 03/23/2017 BS (Cryovial resuspended in 5 mL HMEM + 5% FBS and sedimented @2600 rpm X 10Min. Pellet resuspended in 5 mL HMEM + 5% FBS.)

Lot#: **NA**

MYCOPLASMA TESTING RESULTS

Test Set up Date: **03/31/17**

DIRECT CULTURE METHOD:
No detectable mycoplasma contamination.

INDICATOR CELL CULTURE ASSAY:
Negative: A reaction with staining limited to the nuclear region, which indicates no mycoplasma contamination.

CONTROLS:
Positive and negative controls were appropriate and verify the conclusion of this test.

Figure S1| Mycoplasma report from Bionique
Mycoplasma report from Bionique Testing Labs confirming no mycoplama contamination in the iPSC line used for the study

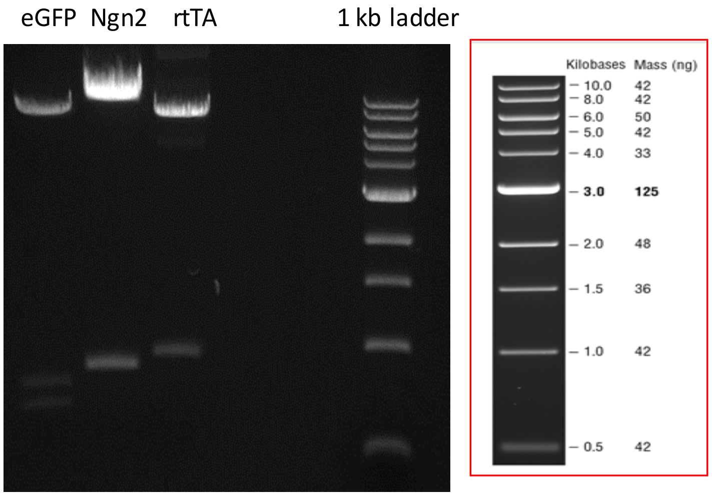

Figure S2 | Restriction enzyme confirmation using Maxiprep RFLP gel electrophoress data of plasmids used in the study

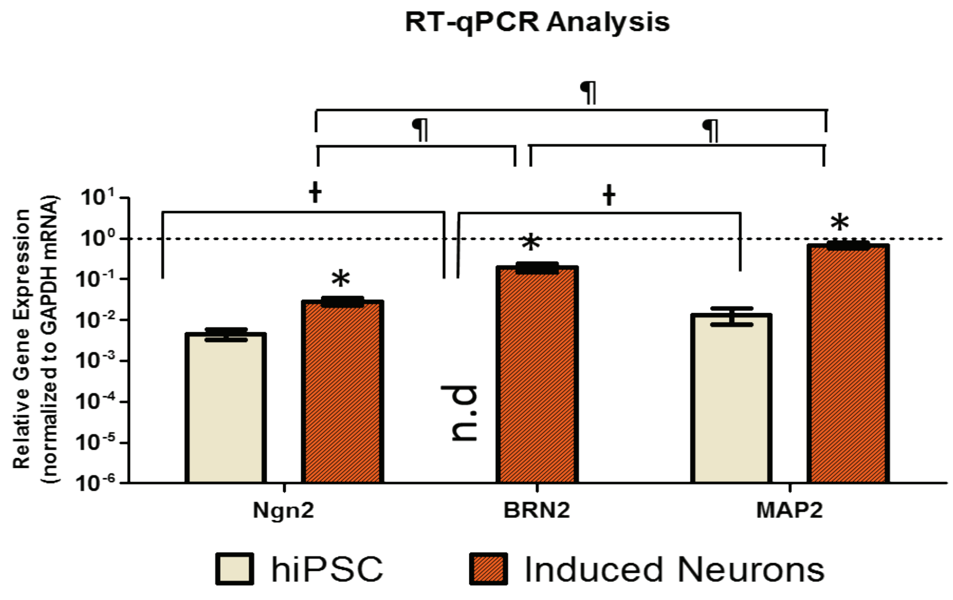

Figure S3 | Gene expression analysis

Comparison of mRNA levels in Cas9-hiPSC and differentiated iNs at day 9, using GAPDH as the internal endogenous control. Levels are expressed in the logarithmic scale. MAP2 expression in iNs is induced ~ 20-fold higher and BRN2 expression is induced > 1,000-fold. Data is represented as average ± stdev (n=4 biological replicates). *=significant increase in expression compared to hiPSC ¶=significant increase in iN group of 1 gene compared to the other gene = significant increase in hiPSC group of 1 gene compared to the other gene

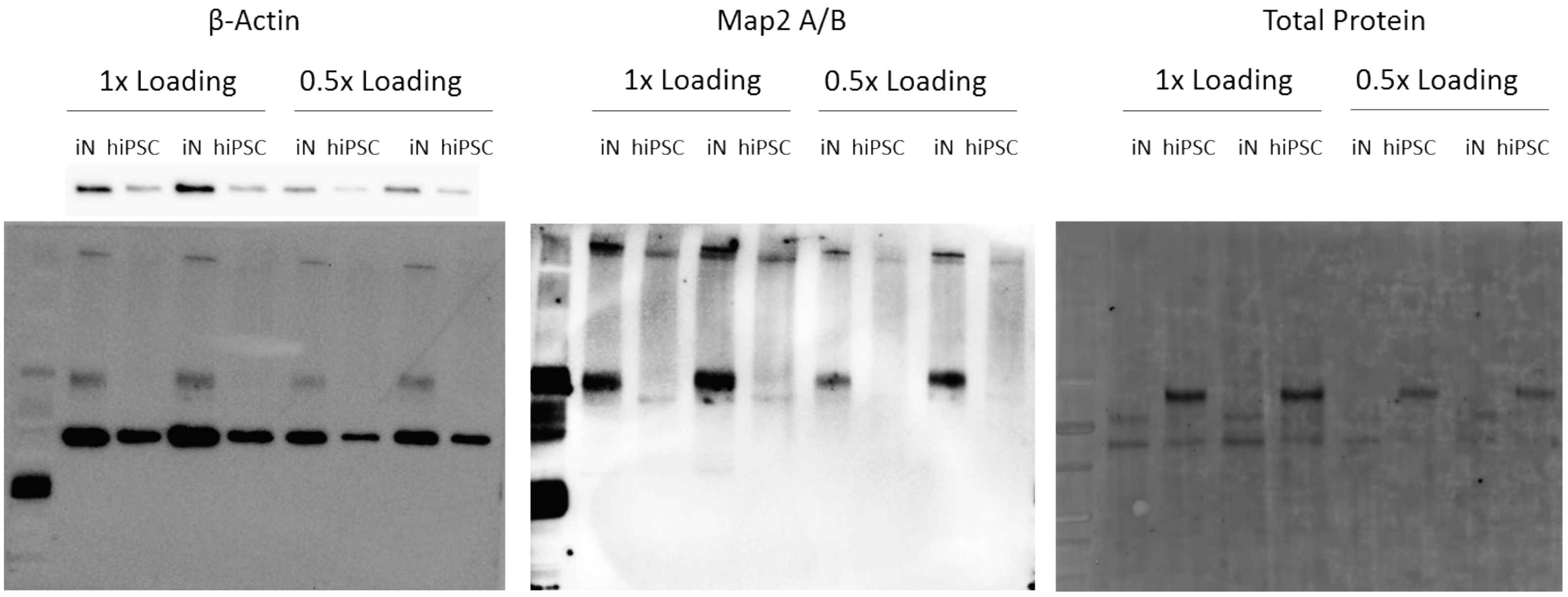

Figure S4 | Characterization of iNs post NGN2-induction

Raw immunoblot membrane demonstrating neuronal marker expression in iNs 6 days post-NGN2 induction. The membranes were cut before probing for anti-Map2 (280 kDa) and anti-β actin(42 kDa) antibodies. Both the control hiPSC and ladder were included in the same gel.

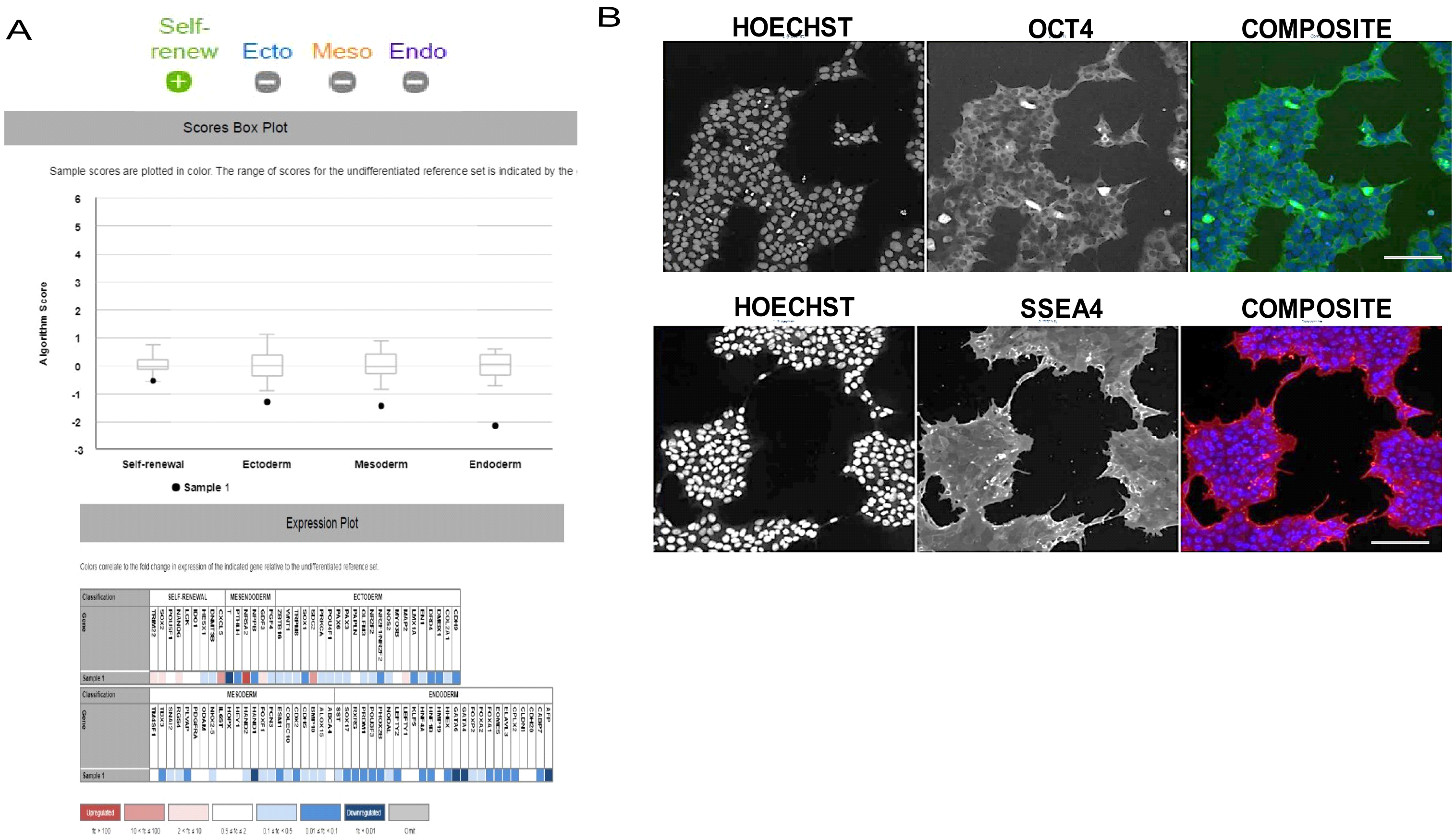

Figure S5 | Characterization of Cas9-hiPSC line.

(A) RT-qPCR scorecard assay of Cas9-hiPSC cell line demonstrating positive for pluripotency markers and negative for other three germ layers (B) Representative images showing immunostaining of Cas9-hiPSC for pluripotency markers, OCT4 and SSEA4. Nuclei were labeled by HOECHST. Scale bar = 100 μm

C

Plasmid	Restric. Enzyme 1 (bp)	Catalog	Restric. Enzyme 2 (bp)	Catalog	Fragment lengths (bp)	Right Plasmid
Tet-O-Ngn2-Puro	EcoR1: 2911	R3101S	Xba 1: 3736	R0145S	825, 9061	✓
FUW-M2rtTA	Nco1: 612	R3193S	Not1: 1524	R3189S	912, 7076	✓
Tet-O-FUW-eGFP	EcoR1: 2818, 3357	R3101S	Pst1: 2183	R3140SS	629, 740, 7746	✓

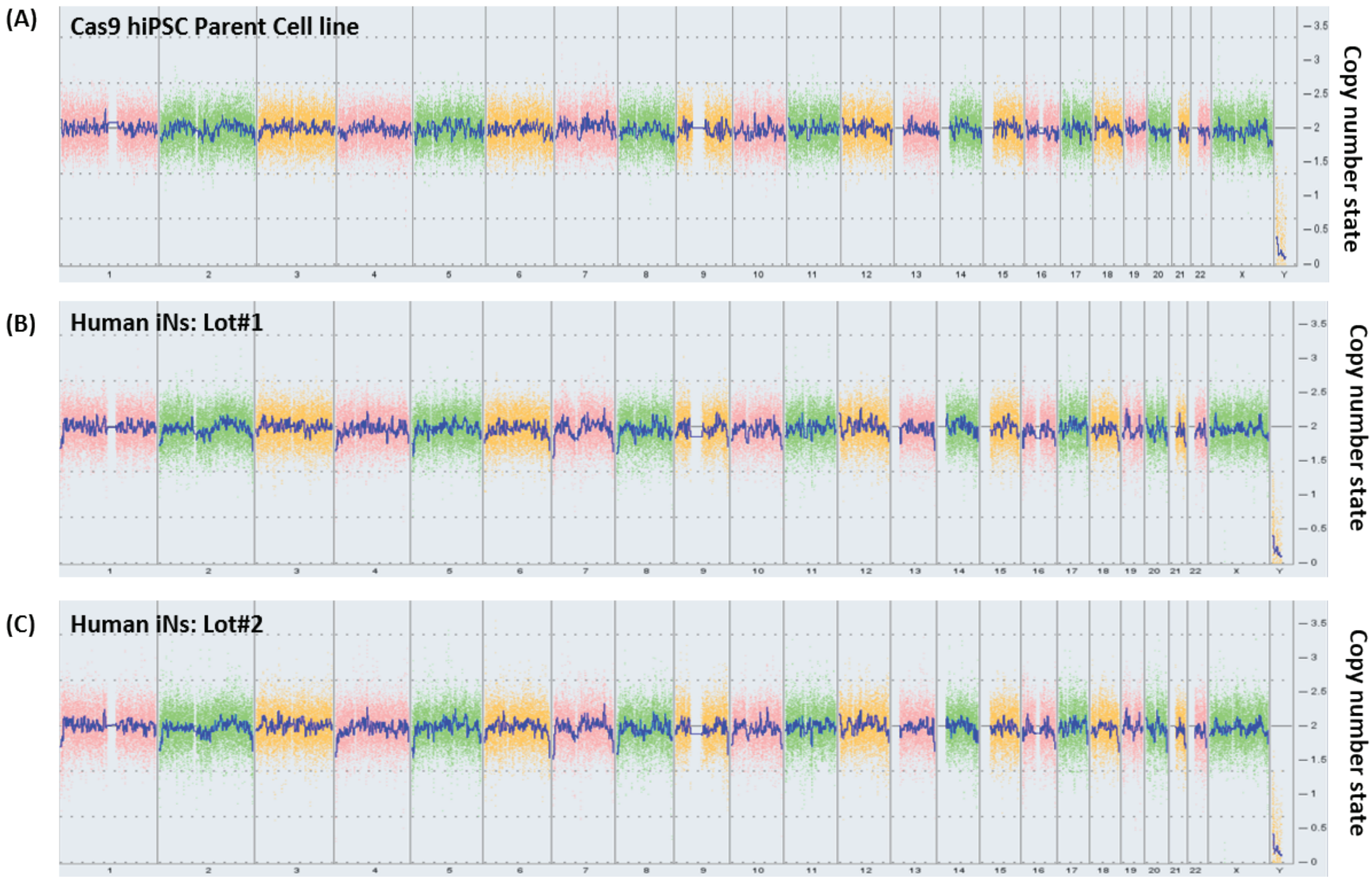

Figure S6 | KaryoStat analysis of iNs

(A) Whole genome view of Cas9 hiPSC parent cell line (Lot# 7765.92) depicting a copy number state of 2 across all chromosomes (except for the Y-chromosome, which is not detected), indicative of a normal female karyotype. (B) and (C) whole genome view of two different lots of iNs that also have a copy number value of 2 across all chromosomes. The whole genome view is shown as Log2 signal intensities (X-axis) of probes on the microarray versus the copy number (Y-axis).

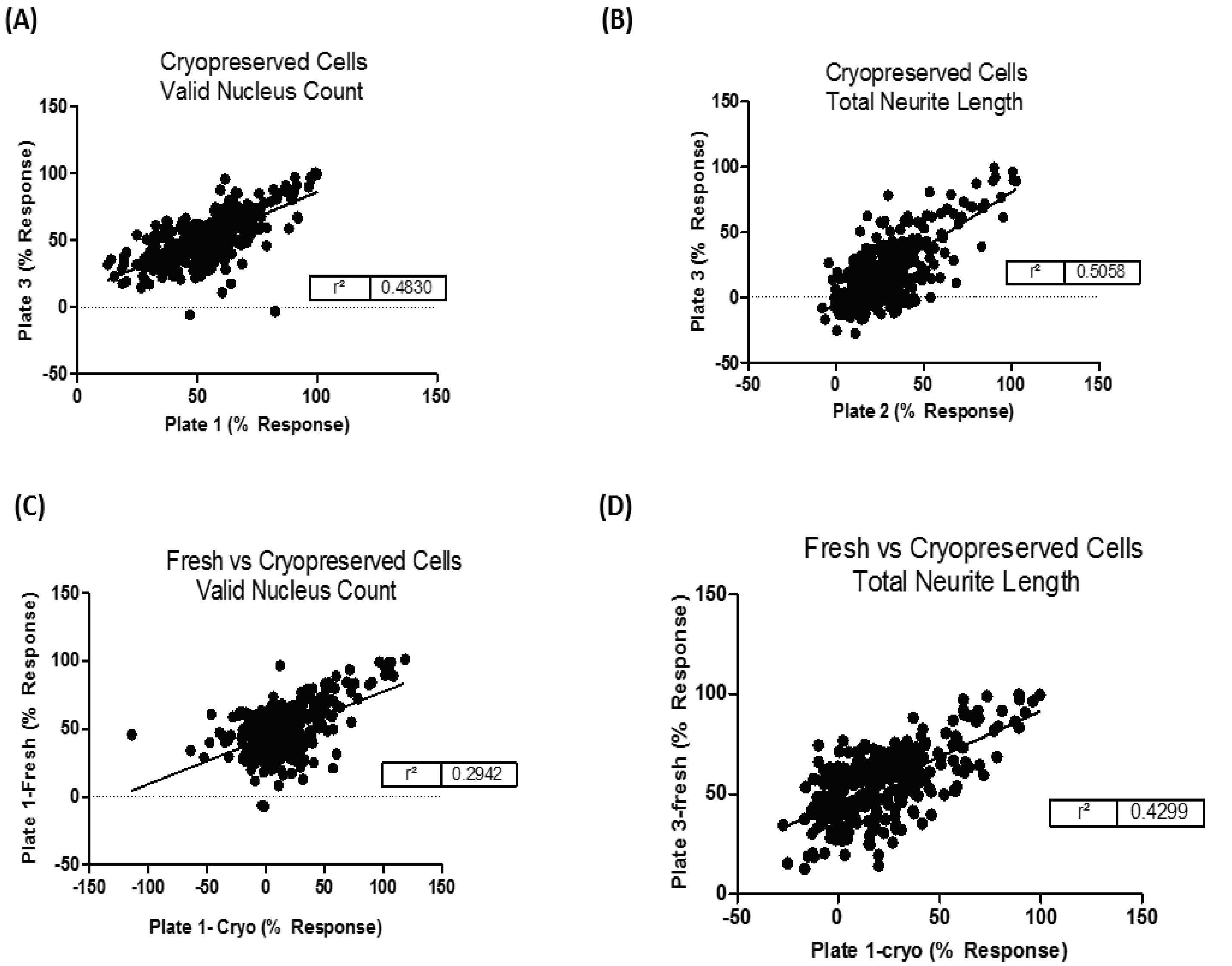

Figure S7 | Addressing robustness of cryopreserved iNs

((A) & (B) Intra-plate correlation plots of the activity found in the LOPAC pilot assays in three different plates for nucleus count and total neurite length. (C) & (D) Correlation plots of the activity found in the LOPAC pilot between freshly differentiated and cryopreserved cells.

Supplementary Table 1 | TaqMan® Gene Expression Assays from Thermo used in this study

Gene	Assay ID
Ngn2	Hs00702774_s1
POU3F2/Brn2	Hs00271595_s1
MAP2	Hs00258900_m1
FOXG1	Hs01850784_s1
GAPDH	Hs02786624_g1

Supplementary Table 2 | Antibodies used in this study

Antibody	Vendor	Catalog	Dilution
1°NeuN	Synaptic systems	101011C5	1:1000
1°MAP2	Synaptic systems	188004	1:1000
1°SSEA-4	Thermo	414000	1:40
1°OCT-4	Thermo	A13998	1:40
2°Goat anti-rabbit Alexa 488	Thermo	A-1108	1:100
2°Goat anti-mouse Alexa 647	Thermo	A-21235	1:100

Supplemental Table 3 | Summary of overall outcome of individual optimization steps

Parameter	Conditions Tested	Observations	Outcome
1. Cell density test (cells/well)	500, 1000, 2500, 5000	Both the 2500 and 5000 cells/well groups yielded CRC curves for BFA	Proceeded with 2500 cells/well to economize cell numbers
2. Length of assay (hrs)	72, 90	Both conditions yielded similar CRC for BFA	Proceeded with final condition of 72 hrs to reduce assay length
3. Reader	EnVision, High content (InCell 6000 and Cellinsight)	1. EnVision detection was not sensitive to neurite length changes 2. InCell 6000 was sensitive but slow, and algorithm set up was tricky. 3. Cellinsight yielded the fastest scan+analysis time and the data matched Incell's data quite closely	Advanced with Cellinsight's Neuroprofiling module
4. Cryopreserved vs. Freshly induced iNs	Cryopreserved iNs were tested vs. freshly differentiated iNs and neurite outgrowth parameters were correlated	Decent correlation for (r^2=0. 42 and 0.39) nucleus count and neurite count between the two conditions. Compound hits from LOPAC plates overlapped between the 2 conditions, which increased confidence in using these cells	Continue using freezer-ready iNs for LOPAC screen
5. Plate coating	Poly-D-Lysine, Poly-L-Ornithine/Laminin	Corning's PLO/Laminin had circular wells that prevented full well analysis using the Cellinsight. Overall, both types of coatings exhibited similar neurite length data for a 72 hr assay	Proceeded with PDL plates since they are more economical than PLO/Lam plates
6. Plate type	Aurora and Greiner: PDL coated plates	Both the plates types showed similar CRC curves	Aurora PDL plates were selected for further optimization but either is fine
7. Environmental conditions	Lab automation incubator, 90 degree manual rotation per day, no rotation in humid chamber	1. Lab automation incubator provided uniform environmental conditions through the length of the assay and was directly coupled to the readouts 2. 90 degree rotation and humid chambers require more manual labor and isn't scalable for large batches	Aurora PDL plates were fitted with a metal lid for compatibility with automation incubator
8. Cellular dispense	Wellmate, FRD, hand plating, magnetic stirbars and direction of dispense	1. Hand-plating caused a mild artifact on some plates. 2. The Wellmate dispenser enabled quick dispense of cells but did not have superior control over the cell number per well 3. FRD dispense was reliable and and fast 4. Magnetic stir-bars and direction of dispense did not have any impact on the assay	FRD was finalized as the cellular dispense automation tool
9. Plate-washing	Biomek, Bluewasher	1. Biomek liquid handler did not remove the staining dye completely and hence elicited high background noise. 2. Did not lose any cells due to centrifugation-based liquid removal by Bluewasher as cells are fixed.	Bluewasher was finalized as the automation tool that will void media and staining solutions
10. High Content objectives	5x , 10x, 20x	1. 5x objective surveyed the entire well and scanned the plates the fastest compared to the other two 2. 10x and 20x needed > 10 FOV that increased scan and analysis time 3. 5x yielded maximum object number necessary to pass Z'	5x Objective will be used for all plates in this assay
11. Control compounds	NGF, Latrunculin (A and B), Blebbistatin, Digitonin, Rotenone, Brefeldin A and Y-27632 compounds were tested for CRC analysis	1. Plating media already contains a cocktail of growth factors so NGF did not induce any significant effect 2. Digitonin, Latrunculin A and B did not yield significant response in the 72 hr assay 3. All other compounds displayed consistent IC50/EC50 over several plates within the 72 hr time	Y-27632, Blebbistatin, Rotenone and BFA were selected as control compounds for assay validation and execution
